# Heat Stress Factors Expressed during Seed Maturation Differentially Regulate Seed Longevity and Seedling Greening

**DOI:** 10.3390/plants9030335

**Published:** 2020-03-06

**Authors:** Concepción Almoguera, Pilar Prieto-Dapena, Raúl Carranco, José Luis Ruiz, Juan Jordano

**Affiliations:** 1 IRNAS, Av. Reina Mercedes 10, 41012 Sevilla, CSIC, Spain; c.almoguera@csic.es (C.A.); ppdapena@irnase.csic.es (P.P.-D.); raulca@cica.es (R.C.); 2 IPBLN, Av. del Conocimiento 17, 18016 Armilla, Granada, CSIC, Spain; joseluis.ruiz@csic.es

**Keywords:** seed maturation, seed longevity, photomorphogenesis, seedling greening, differential regulation, HSF

## Abstract

Heat Stress Factor A9 (A9), a seed-specific transcription factor contributing to seed longevity, also enhances phytochrome-dependent seedling greening. The RNA-seq analyses of imbibed-seed transcripts here reported indicated potential additional effects of A9 on cryptochrome-mediated blue-light responses. These analyses also suggested that in contrast to the A9 effects on longevity, which require coactivation by additional factors as A4a, A9 alone might suffice for the enhancement of photomorphogenesis at the seedling stage. We found that upon its seed-specific overexpression, A9 indeed enhanced the expected blue-light responses. Comparative loss-of-function analyses of longevity and greening, performed by similar expression of dominant-negative and inactive forms of A9, not only confirmed the additional greening effects of A9, but also were consistent with A9 not requiring A4a (or additional factors) for the greening effects. Our results strongly indicate that A9 would differentially regulate seed longevity and photomorphogenesis at the seedling stage, A9 alone sufficing for both the phytochrome- and cryptochrome-dependent greening enhancement effects.

## 1. Introduction

In plants, the heat-shock response and some crucial developmental processes are regulated by a gene family of transcription factors known as the heat-shock transcription factors (HSFs). The functional specialization of the multiple plant HSFs is, for the most part, unexplored. Only HSFs from a few plant species have been functionally analyzed [1,2]. Our lab has characterized HSFs, which, in sunflower (*Helianthus annuus* L.), contribute to longevity, thermotolerance, and desiccation tolerance of seeds. HaHSFA9 (A9; *Helianthus annuus* Heat Stress Factor A9) is a peculiar Class A HSF that, in sunflower, is expressed only in seeds [3]. The gaining of function upon the overexpression of A9 in transgenic tobacco has indicated its involvement in thermotolerance, seed longevity, and tolerance to extreme desiccation [4,5]. A9 activates a genetic program—the “A9-programme”—that includes subsets of genes encoding Heat Shock Proteins (HSP) normally expressed during zygotic embryogenesis in seeds. Furthermore, the photosynthetic apparatus and green organs of 35S:A9 seedlings (constitutively overexpressing A9) showed an unusual resistance to extreme conditions of dehydration and oxidative stress [6].

In connection with seed longevity, we reported some requirements and consequences of loss-of-function of A9. Different modified forms of A9, expressed under the seed-specific DS10 promoter, were analyzed in transgenic tobacco seeds. Transcription-inactive forms of A9 (as A9M1) were inefficient compared to an active repressor form (A9 fused to SRDX, A9M3). Thus, using only A9M3, we observed a substantial reduction in seed longevity [7]. This strongly indicates that A9 is not the sole Class A HSF involved in transcriptional activation of the “A9-programme” in developing seeds. Subsequently, HaHSFA4a (A4a; *Helianthus annuus* Heat Stress Factor A4a) was identified as one of such accessory HSFs [8]. Interestingly, both A4a and A9 were repressed by the auxin/Indole-3-Acetic Acid (aux/IAA) protein HaIAA27, which revealed a connection between seed longevity and auxin signaling: aux/IAA proteins reduced seed longevity by interfering the A9-A4a synergic interaction [8,9]. A9 and A4a coactivate the same genetic program involving specific sHSP target genes. This has been confirmed by observing enhanced seed longevity in DS10:A4a and DS10:A9/A4a transgenic tobacco lines, which specifically overexpress A4a, or A4a with A9, in seeds [10]. Similar analyses with 35S:A4a and 35S:A9/A4a lines revealed enhanced tolerance to vegetative severe dehydration and oxidative stress in young transgenic seedlings, furthermore showing that A4a strictly requires A9 to cause the enhanced stress resistance [10].

Plants use sunlight as an important developmental cue. Chloroplast biogenesis starts, for the first time, during plant embryogenesis, normally halts during seed development, and continues after germination. Embryos in seeds contain immature plastids (proplastids) that, during dark germination, develop into partially assembled plastids that completely transform into chloroplasts only after photomorphogenesis is induced by light [11]. Light perception by different receptors is crucial for the initiation and progression of photomorphogenic development. This includes the receptors for far-red (FR) and red (R) light, which are Phytochrome A (PHYA), and Phytochrome B (PHYB), respectively (see the reviews [12,13,14]). The FR and R wavelengths of white light suffice—in separate—for photomorphogenesis. However, plants also use different receptors for blue light, including Cryptochromes (CRY) and Phototropins (PHOT), respectively reviewed in [15,16]. A recent publication from our lab also demonstrated a functional link between A9 and the initiation of seedling photomorphogenesis [17]. This link is active under darkness immediately after seed germination, and also upon exposure to light, partially operating through direct and indirect effects on the PHYA and PHYB photoreceptors. In transgenic tobacco plants, A9 thus causes complex effects, resulting in accelerated photomorphogenesis. This adds to the enhanced drought, heat, and oxidative stress tolerance also conferred by A9, as revealed by our former studies [4,5,6]. However, it has not yet been explored whether A4a coactivates the photomorphogenic effects induced by A9 in a similar way to that reported for the effects of A9 on seed longevity. In this work, we performed comparative RNA-seq analyses of the overexpression effects of A9 and A4a. We also reported comparative loss-of-function analyses of seed longevity and seedling greening, performed using dominant-negative and inactive forms of A9. Collectively, our results support a model whereby A9 enhances seedling photomorphogenesis without the concourse of additional seed HSFs as A4a.

## 2. Results

**Figure 1:**
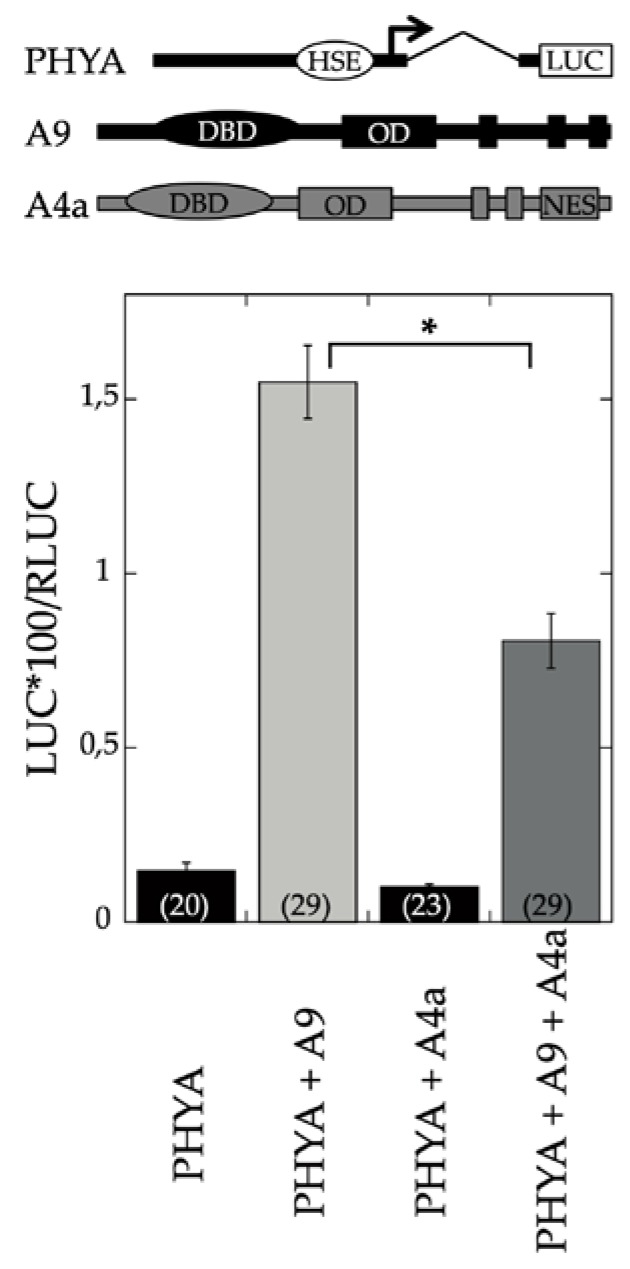
A4a impaired the transient activation of the PHYA promoter by A9. Top: Illustration showing the PHYA: LUC reporter gene and the A9 and A4a effectors used in the transient expression assays (genes are not drawn to scale). LUC: Luciferase reporter gene, DBD: DNA Binding Domain, OD: Oligomerization Domain, NES: Nuclear Export Signal. HSE (Heat Shock *cis*-Element) denotes the imperfect *cis-*elements used by A9 in *NtPHYA1.* Bottom: transient expression assays. Numbers in parentheses show the number of replicates for each reporter/effector plasmid combination. The asterisk indicates a statistically significant difference (see Table S7). Error bars denote the SEM.

**Figure 2:**
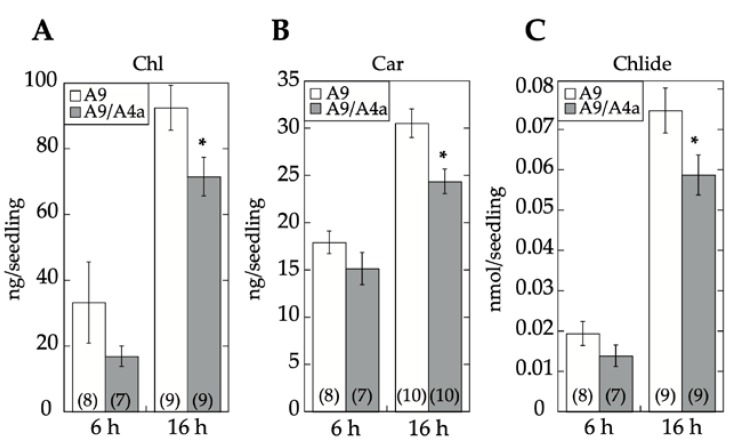
A4a reduced greening in the A9 genetic background. Total chlorophyll (**A**), carotenoid (**B**), and chlorophyllide (**C**) content in sibling 35S:A9/A4a (A9/A4a) and 35S:A9 (A9) seedlings. Seeds from the different sibling lines were germinated and kept under darkness for 4 days. After the germination treatment, seedlings were transferred to continuous white light conditions for 6 h or 16 h, as indicated. The average photopigment content ± SEM was then determined in at least three experiments, performed with three different pairs of sibling lines. The asterisks denote significant statistical difference (*p* < 0.05; see Table S7). Numbers in brackets denote sample size (number of Petri dishes, each dish providing average measurements from 30–60 seedlings).

A9 is a seed-specific transcriptional activator [3] that enhances gene expression connected both with seed longevity [4] and seedling photomorphogenesis [17]. To investigate whether the regulation by A9 is similar or different in both cases, we first analyzed the effect of A4a on transient transcriptional activation of the PHYA promoter, a photoreceptor that is directly activated by A9 [17]. The results depicted in Figure 1 showed that A4a attenuated the transcriptional activation of PHYA by A9. Additional experiments demonstrated the inhibitory effects of A4a on seedling photomorphogenesis in the A9 genetic background, for example, in experiments of early greening under white light (Figure 2). These results contrast the published effects of A4a- on A9-induced seed longevity and on promoters of genes (as sHSP) involved in seed longevity, where A4a functions as an A9-dependent synergic transcriptional coactivator [10], which enhances seed longevity, as determined by controlled-deterioration assays [4]. The results in Figure 1 and Figure 2 indicate that the A9 effects on seed longevity and seedling greening were differentially regulated.

### 2.1. Transcriptomic Analysis of the Effects of A9 and A4a

**Figure 3:**
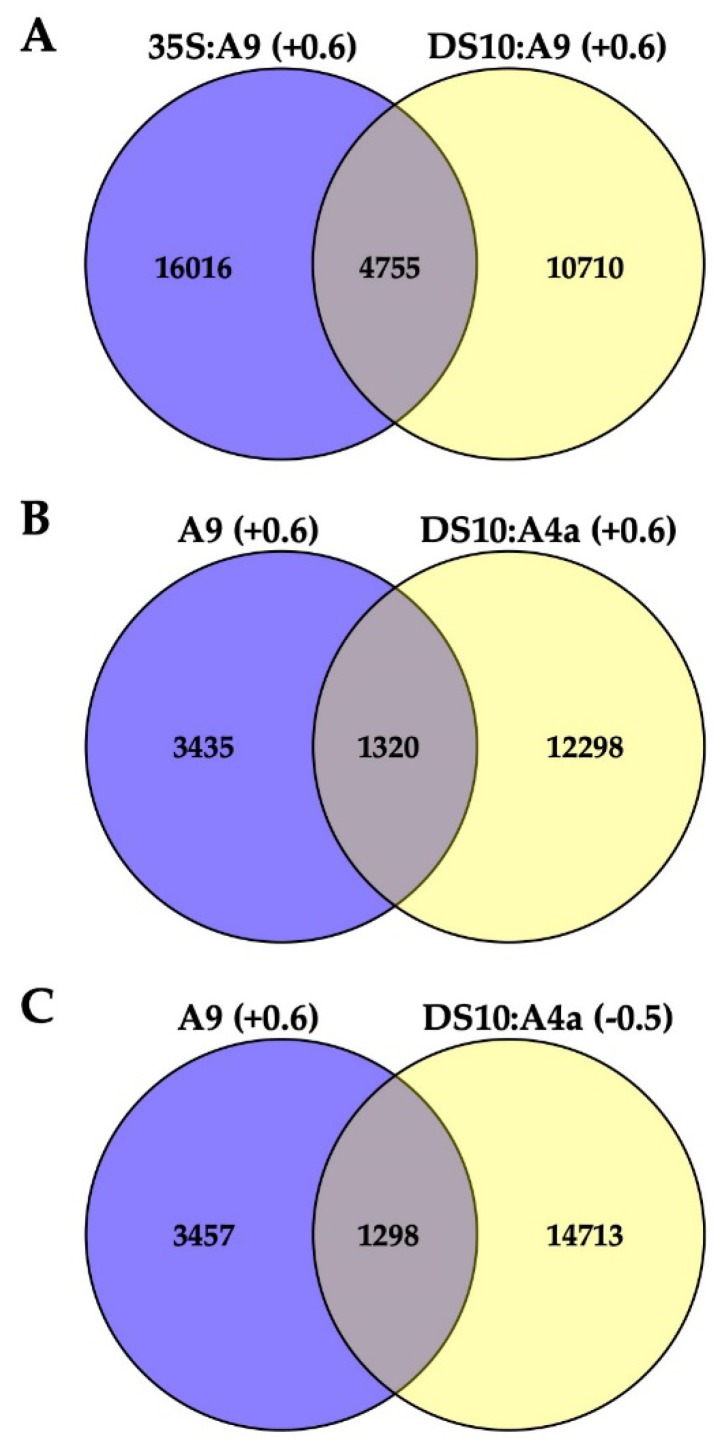
Comparisons of differentially regulated genes from the different RNA-seq analyses. (**A**) The A9up set of 4755 genes consistently upregulated in the 35S:A9 and DS10:A9 RNA-seq experiments. (**B**) Comparison of the A9up genes with genes upregulated in the DS10:A4a RNA-seq. (**C**) Comparison of the A9up genes with genes downregulated in the DS10:A4a RNA-seq. The considered log2fold upregulation (+0.6) or downregulation (−0.5) level is indicated for each comparison. See the text in the Results section for further explanation and interpretation of the different gene subsets obtained from the RNA-seq comparisons summarized here and listed in Tables S1 and S3–S6. Examples for the different gene subsets mentioned in the text are included in Table S2.

The differential regulation indicated by the results of Figure 1 and Figure 2 was further investigated by exploratory (pooled, single-replicated; see Materials and Methods for details) RNA-seq analyses of transcript accumulation induced by A9 or A4a overexpression in transgenic tobacco. Each transgene in the various lines was integrated at different single locations (as detailed in Materials and Methods). For RNA-seq, we used the transgenic A9 lines and sibling nontransgenic (NT) segregant lines that were described by our lab in precedent works [4,5]. For A9-induced (A9up) transcripts, we identified mRNAs that were consistently upregulated in both seedlings of 35S:A9 lines and in imbibed seeds of DS10:A9 lines (by comparison with the respective NT sibling lines in each case). The effects of A4a were determined using imbibed seeds (therefore expressing endogenous A9) from the sibling DS10:A4a and NT lines [10] because we previously determined that A4a was not able to activate transcription in absence of A9, for example, in the vegetative organs of 35S:A4a plants [10]. Table S1 includes a total of 4755 identified genes consistently upregulated by A9 at least 1.5-fold (Log2 fold ≥ 0.6; the A9up set) in both the 35S:A9 and DS10:A9 RNA-seq experiments (Figure 3A). The A9up set included the target genes of A9, which, in connection with seed longevity (for example, multiple sHSP genes), or photomorphogenesis (for example, PHYA, PHYB, and ELONGATED HYPOCOTYL 5, HY5) were confirmed by RT-qPCR and with other different analyses in our precedent publications [4,5,7,9,10,17]. This supports the quality of our exploratory RNA-seq. Table S2 outlines some examples of these important genes. The sufficient quality of the RNA-seq analyses used in Figure 3 for the inference of differential expression and regulation of putative A9 target genes is also supported by functional verification of predictions from Figure 3 (see below and the Discussion section).

Figure 3B,C represent data of the DS10:A4a RNA-seq experiment. Figure 3B shows that the set of 13,618 A4a-upregulated genes (listed in Table S3) comprehended a subset of the A9up set (the 1320 genes listed in Table S4) that included genes known from our previous studies to be coactivated by A9 and A4a, for example, seed sHSP genes expressed in connection to longevity [4,9,10], namely HSP18.2 CI, HSP22.0 CIV, and HSP26.5 M (see Table S2). Figure 3C shows that the set of 16011 A4a-downregulated genes (listed in Table S5) included 1298 A9up genes. This subset of 1298 A9up genes (listed in Table S6) included A9 targets connected to the published PHYA- and PHYB-dependent photomorphogenic responses enhanced by A9 [17]. Among these genes, we outline HY5, a transcription factor acting as the regulatory hub for the responses to visible (far-red, red, blue) and UV-B light. Coactivation by A9 and A4a of sHSP genes involved the simultaneous binding to noncanonical HSE (Heat Shock *cis* Element) of the two factors, most likely bound as an A9-A4a heteromultimer [8,18]. The 1298 A9up genes identified in the downregulated set might represent genes (such as HY5) activated by A9 alone, which bound to different noncanonical HSE as homomultimers (see the Discussion section). The results of Figure 1 and Figure 2 and the RNA-seq results summarized in Figure 3B,C encouraged us to perform additional experiments for the functional confirmation of our hypothesis that A9 target genes involved in enhancing seedling greening would be regulated mainly by A9 alone in contrast to the reported coactivation by A9 and A4a of the A9 target genes involved in seed longevity [10].

### 2.2. A9 Enhances Responses to Blue Light Mediated by CRY1

**Figure 4:**
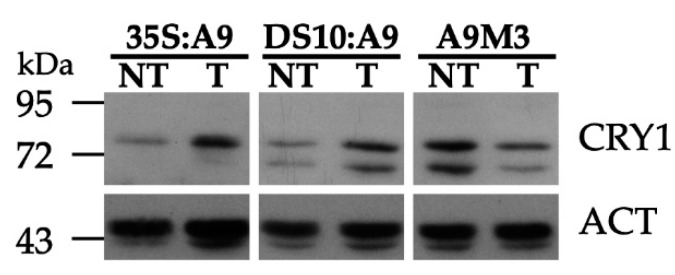
A9 enhanced the accumulation of the CRY1 protein in transgenic tobacco. Detection of CRY1 antibody-reacting proteins in total protein samples from imbibed seeds and seedlings grown under darkness. Results for 10-day-old seedlings illustrated with samples from a representative sibling pair of nontransgenic (NT) and transgenic (T) (35S:A9) lines. Results for seeds imbibed for 24 h depicted for representative pairs of NT and T (DS10:A9 or DS10:A9M3) lines. Equal loading of total protein per lane was verified with antibodies against actin (ACT).

The A9up set genes allowed us to predict the additional effects of A9, such as the enhancement of cryptochrome-dependent blue-light responses indicated by the presence of numerous CRY1/HY5 targets, for example, the sigma factor SIG1, 9-cis-epoxycarotenoid dioxygenases (NCED), chalcone synthase (CHS), dihydroflavonol-4-reductase (DFR), and leucoanthocyanidin dioxygenase (LDOX), However, the A9up set did not include CRY1 itself (see Table S1 and the gene examples listed in Table S2). Precedents in the literature showed that polyclonal antibodies against a 17 kDa C-terminal fragment of the *Arabidopsis thaliana* CRY1 protein specifically detected the *Nicotiana tabacum* CRY1 protein [19]. We therefore used these antibodies to investigate whether A9 enhanced CRY1 protein accumulation in our transgenic A9 lines. The results in Figure 4 indeed show that A9 enhanced the accumulation of a protein band matching the expected mobility of the *Nicotiana tabacum* CRY1 protein. This was observed with samples prepared from dark-imbibed DS10:A9 seeds, and also with samples from dark-grown 35S:A9 seedlings. These results suggest that A9 might increase CRY1 protein accumulation. The enhancement by A9 of CRY1 protein accumulation was confirmed by similar analyses performed with samples of imbibed seeds from loss-of-function DS10:A9M3 lines. The expression of a dominant-negative form of A9 in these lines caused the converse effect, decreasing the accumulation of the proteins detected with the anti-CRY1 antibodies (see also Figure 4).

**Figure 5:**
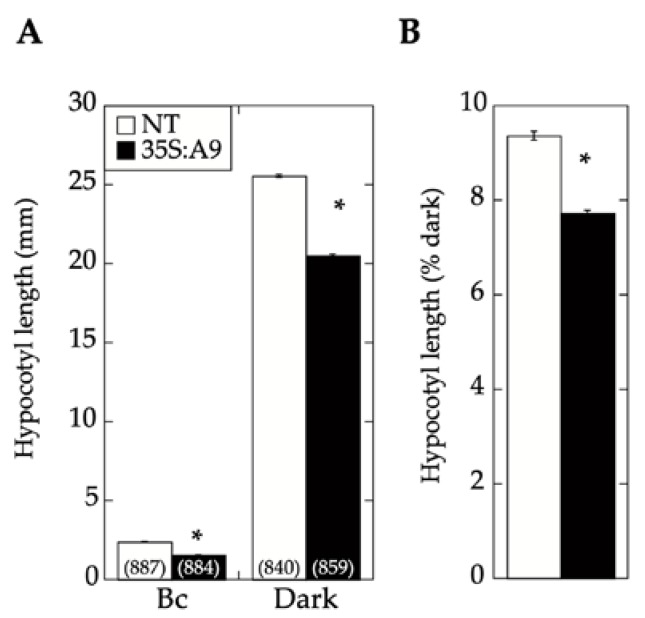
CRY1-dependent hypocotyl growth responses to blue light were enhanced by A9. Hypocotyl growth inhibition of 35S:A9 seedlings after illumination with blue light. (**A**) Hypocotyl length was measured in seedlings grown under darkness for 3 days and then illuminated under continuous monochromatic blue light (Bc) at a fluence rate of 20 μmol m^−2^. (**B**) The measured hypocotyl lengths from panel A are represented as the percentage of length measured after illumination with respect to that in dark-grown seedlings. This eliminated the differences between NT and 35S:A9 seedlings observed without illumination and allowed us to assay only the effect on the blue-light response. Note that in tobacco seeds, after 3 days of imbibition under darkness, growth was only detectable as the root point started to protrude the seed coat. Three pairs of 35S:A9/NT sibling lines were analyzed in at least two independent experiments. Numbers in brackets represent the number of measurements. Asterisks indicate the statistically significant differences (see Table S7). Error bars denote the SEM.

The A9-enhanced perception of blue light mediated by CRY1 (as inferred from Figure 4) was finally confirmed with functional hypocotyl-elongation assays. In the experiments summarized in Figure 5, monochromatic blue light was used at a fluence, which, in previous studies, was shown to mainly affect light perception by CRY1 [20]. Under these blue-light illumination conditions, the expression of A9 in the 35S:A9 seedlings caused a significant reduction of hypocotyl growth compared to that of NT sibling seedlings. Therefore, A9 enhanced the reduction of hypocotyl growth in response to blue light in addition to the similarly demonstrated enhancement of responses to red and far-red light [17]. As discussed in our former study [17], the expression window in the A9 loss-of-function lines did not allow their use for confirmation of the results in hypocotyl-elongation assays.

### 2.3. Comparative Loss-of-Function Effects of A9 in Seed Longevity and Seedling Greening

We investigated the suggested differential regulation by A9 of seed longevity and seedling greening by comparing the effect of loss-of-function caused by two different forms of A9: A9M1 and A9M3. We previously used this strategy to analyze the effect of A9 loss-of-function on seed longevity [4]. The A9M1 form is transcriptionally inactive because of a deletion in the C-terminal region of A9, which includes three AHA motifs (enriched in aromatic, hydrophobic, and acidic amino-acid residues) that are required for transcriptional activation [1,21]. In the A9M3 form, A9 is fused to a potent SRDX repressor domain causing dominant-negative effects [7,22]. Seed longevity was assessed by two different protocols of artificial aging: (a) BTA, a fast-deterioration protocol (a seed fast-deterioration protocol based in a Basal Thermotolerance Assay) and (b) by a standard Controlled seed Deterioration Treatment (CDT) [7]. Regarding longevity, in our former study, we only compared the DS10:A9M1 and DS10:A9M3 transgenic lines with their respective transgenes integrated as single events in heterozygosis. The results of BTA assays showed that a substantial reduction of seed-longevity was observed only with the DS10:A9M3 seeds, compared to much lower effects seen with the DS10:A9M1. Loss-of-function effects on seed-longevity and on gene expression associated with seed longevity were confirmed with homozygous DS10:A9M3 transgenic lines using CDT [7,23].

**Figure 6:**
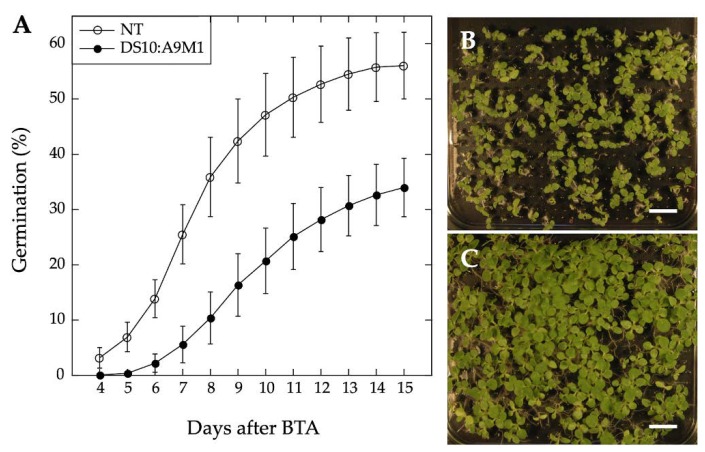
A9 loss-of-function effect on seed deterioration in the DS10:A9M1 homozygous lines. (**A**) Results of Basal Thermotolerance Assay (BTA) assays performed with seeds from three different DS10:A9M1 lines compared with the sibling NT lines. Average germination percentages at different times after BTA (for a total of three independent experiments per line) are shown. Representative photographs of DS10:A9M1 (**B**) and sibling NT seed survival (**C**) 15 days after BTA in both cases. Scale bars: 1 cm. The observed seed longevity reduction for DS10:A9M1 was moderate and statistically significant, as expected from our precedent analyses of heterozygous lines (see Figure 2A in [7]). However, the observed reduction was lower than what previously reported for homozygous DS10:A9M3 seeds (see Figure 3 in [7]). Furthermore, statistics confirmed that the effects of DS10:A9M1 and DS10:A9M3 on longevity in homozygous seeds were highly significantly different (for statistics, see Table S7).

**Figure 7:**
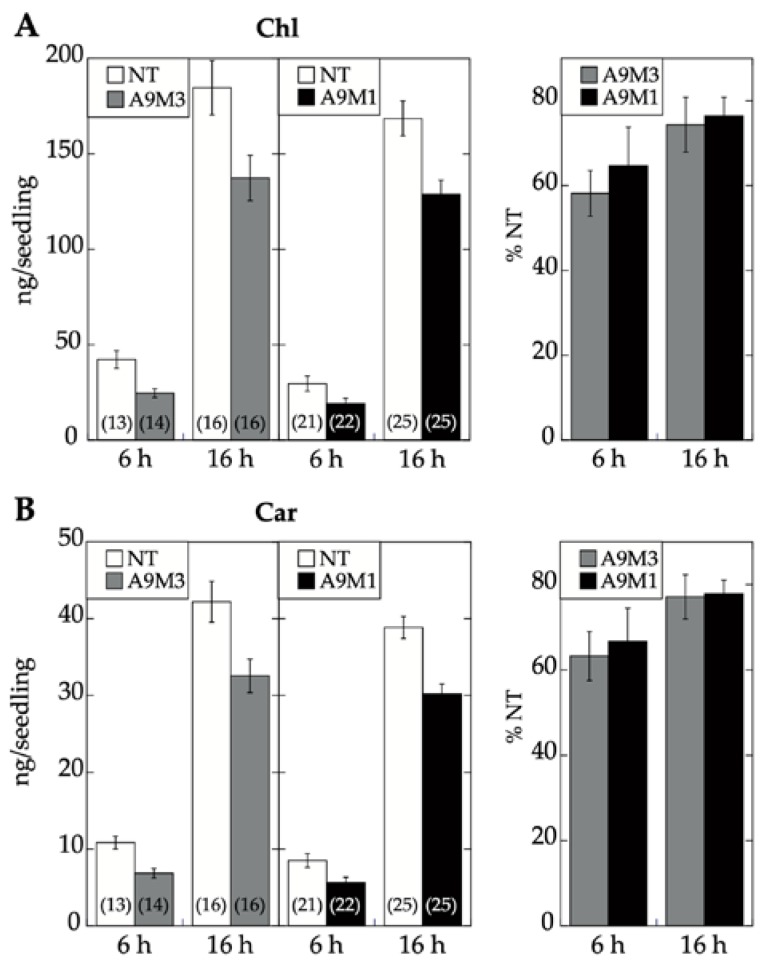
Similar A9 loss-of-function effect on seedling greening in the DS10:A9M1 (A9M1) and DS10:A9M3 (A9M3) homozygous lines. Seeds from the different transgenic (A9M1 or A9M3) and the corresponding sibling NT lines were germinated and kept under darkness for 4 days. After dark germination, seedlings were transferred to continuous white-light conditions for 6 h or 16 h, as indicated, before pigment extraction and quantification. (**A**) Comparison of greening reduction using total chlorophyll content. (**B**) Comparison of greening reduction using total carotenoid content. Average photopigment content was determined and depicted as in Figure 2. Percent pigment content with respect to NT is provided in the panels to the right in each case. Statistical analyses did not find significant differences between pigment content reduction observed in the A9M1 and A9M3 seedlings after either 6 h or 16 h illumination (see Table S7). Data from at least three experiments, performed with two different pairs of sibling lines. Error bars denote the SEM.

We used homozygous DS10:A9M1 transgenic lines and BTA assays to verify the published loss of function effects in connection with seed longevity. Regarding the A9 effects on seedling greening, we used the DS10:A9M1 and DS10:A9M3 homozygous lines for the comparative loss-of-function. As expected, seed longevity was decreased to some extent in the homozygous DS10:A9M1 seeds. However, these seeds still survived BTA deterioration significantly better than previously reported for homozygous DS10:A9M3 seeds (respectively, Figure 6 and [7]). These results confirmed that, regarding longevity, a loss-of-function effect could only be efficiently obtained with A9M3, in which A9 is converted into a very strong active repressor that has dominant-negative transcriptional effects not only on A9, but also on similar class A HSFs, such as A4a [7,22]. The loss-of-function of seedling greening enhancement by A9 was analyzed in a similar way, using the white light-induced greening conditions in our previous study [17]. Seedling greening and photomorphogenic development was quantitatively analyzed by chlorophyll and total carotenoid pigment determinations (respectively in Figure 7A,B). The results, supported by statistical analyses in Figure 7, clearly showed that regarding seedling greening enhancement determined after 6 h and 16 h of illumination, loss-of-function caused by A9M1 did not significantly differ from that caused by A9M3. Therefore, both forms were similarly efficient. These results contrast the observations regarding loss of function of seed longevity ([7] and Figure 6), supporting that the reported enhancement of seedling greening [17] is caused mainly by A9, thus not requiring additional HSFs (such as A4a for seed longevity, [4]).

## 3. Discussion

Seed longevity, desiccation tolerance, and seedling performance are important biological processes targeted by plant biotechnology [24,25,26]. A9, a seed-specific transcription factor, participates in controlling seed longevity and embryo desiccation tolerance [3,4,5,7,9]. A9 also contributes to the acceleration of early seedling greening. This greening effect occurs shortly after germination and seedling emergence (before the A9 protein accumulated during seed maturation is degraded) [17]. Seedling greening acceleration impacts a limiting step for establishment, as it facilitates the transition from heterotrophic (dependent on seed reserves) to autotrophic (photosynthetic) growth [11,26]. Collectively, our results support a model where A9 alone enhances seedling photomorphogenesis without the concourse of additional seed HSFs. This contrasts the enhancement of seed longevity, where A4a is also required as a coactivator of A9 [7,10]. Our model of differential regulation is strongly supported by differential loss-of-function results, showing that inactive A9 (A9M1) sufficed for a negative effect on greening, but not on longevity (Figure 6 and Figure 7, see also [7]). These results, in turn, agree with the puzzling negative effect of A4a on the activation of PHYA promoter and on greening (Figure 1 and Figure 2), and with the inference from the comparative RNA-seq analyses, where the accumulation of a subset of transcripts from genes activated by A9 (including the crucial light-response hub regulator HY5 and blue-light responsive genes as CHS, FLS, LDOX, DFR, and NCED1) are negatively affected by A4a (Figure 3C, Tables S2 and S6). Upon A4a overexpression, competition between inactive A4a homomultimers and endogenous A9 might be the cause of the observed transcript downregulation. This interpretation agrees with and also explains the results in Figure 1 and Figure 2. Thus, A4a, which, by itself, lacks a transcriptional activity *in planta* [8,10], would compete for A9, causing all the observed negative effects.

The mostly nonredundant (sufficient) effect of A9 on seedling greening might facilitate some future technological applications, as, for example, the engineering of improved seedling establishment [26]. For A9 to be used with this purpose in the desired crop(s), only the functional conservation of the crop A9(s) would be required, which might occur, at least, in most dicot plants (but not in monocot plants lacking orthologous A9s [1,2]). In contrast, using A9 for engineering-enhanced seed longevity would additionally require the functional conservation of the crop A4a(s). Plant A4a(s) have been shown to have functional differences [27,28,29,30]. In addition, the lack of intrinsic transcriptional activity *in planta* has been reported only for sunflower A4a [8,10], which limits the use of A9 for enhancing seed longevity to plants similar to sunflower and tobacco (*Asteridae*), where the peculiar A4a properties and a synergic functional interaction with A9 is known to be conserved [10].

The here reported A9 RNA-seq data (Figure 3A, Table S1) allowed, after subsequent molecular (Figure 4) and functional assay confirmation (Figure 5), extending the embryonic A9 link to effects on the blue-light receptor CRY1. Thus, A9 would link seed maturation and seedling photomorphogenesis by acting on photoreceptors for light with wavelengths placed at both ends of the visible spectrum: CRY1 (for blue light; as confirmed by the results in Figure 4 and Figure 5), in addition to PHYA (for far-red light) and PHYB (for red light) as reported earlier [17]. Tables S1 and S2 (where some important gene examples are outlined) indicate the potential upregulation of additional light receptors, including different phytochromes (PHYC for red light, [12,13,14,31]) and phototropins (PHOT2 for blue light, [16]). In connection with early photomorphogenesis and seedling establishment, responses to blue light mainly mediated by CRY1 (and also by PHOT) receptors are crucial for inducing massive gene expression changes that occur after extensive chromatin and nuclear architecture reorganization [32,33]. As A9 indeed enhanced a CRY1-dependent response (Figure 5), it is perhaps not surprising that Table S1 includes multiple A9-upregulated genes connected with histone modification and chromatin remodeling: for example, histone chaperones ASF1A and ASF1B, mediator subunit MED18, histone deacetylase HDAC2, and DNA Helicase CHR10 (Table S2). This suggests a potential impact of A9 on these processes, at least in part mediated by A9 effects on CRY1 and PHOT receptors. This suggestion is currently under investigation in our lab. The results reported here demonstrate that the A9up dataset (Figure 3A, Table S1) can be used to predict novel useful effects of A9, and the gene subsets listed in Tables S4 and S6 can be used to predict a regulation, respectively, involving A9 and A4a (for seed longevity), or mainly A9 (for seedling greening), which, in turn, could have implications on the understanding and future practical application on these novel effects.

## 4. Materials and Methods

### 4.1. Plant Material and Transgenic Lines

Tobacco (*Nicotiana tabacum* L. var. Xanthi) was used for all experiments. The sibling pairs of nontransgenic and transgenic lines used in this report were obtained after segregation and propagation from single-integration homozygous lines, described in our precedent publications. Each transgene was integrated at different genomic locations. The material used for RNA-seq included three different pairs of sibling 35S:A9 and NT lines [5], sibling DS10:A9 and NT lines [4], and sibling DS10:A4a and NT lines [10]. For the greening experiments in Figure 2, we also used three sibling pairs of homozygous double transgenic 35S:A9/A4a and single transgenic 35S:A9 lines propagated from material obtained as previously described [10]. For the experiments of comparative loss-of-function (Figure 6 and Figure 7), we used material propagated from two different pairs of sibling DS10:A9M3 and NT lines [10]. From the described heterozygous sibling DS10:A9M1 and NT material [7], and as described for the rest of the homozygous transgenic material [4,5,7,10], we also obtained three different pairs of sibling homozygous DS10:A9M1 and NT lines, which were also used for the experiments in Figure 6 and Figure 7. Seed sterilization, germination, and seedling growth under controlled photoperiodic illumination conditions of 16 h light/8 h dark were as previously described [17].

### 4.2. Promoter Activation Assays

Transient expression was performed 24 h after bombardment of sunflower leaves and analyzed essentially as previously described [17]. Dual Luciferase assays were performed with the Promega system (Promega Corporation, Madison, USA) according to the manufacturer’s instructions. The bombarded sunflower leaves were homogenized in 150 μL of Passive Lysis buffer. After centrifugation for 7 min at 12,000× *g* and 4 °C, we assayed 2 μL of each extract in 40 μL of Luciferase Assay reagent (LAR II). For the Renilla luc assays (Rluc, internal reference), we added 40 µL of 1 × Stop and Glo solution. Luc an Rluc activities were sequentially measured in TD-20/20 luminometer. The amounts of plasmid DNA (per DNA precipitate, used for five shots) were 0.25 μg of pBI221-HaHSFA9, 5 μg of pBI221-HaHSFA4a (the effector plasmids), 5 μg of the PHYA:LUC reporter plasmid [17], and 1 μg of pBI221-Rluc, the internal reference plasmid.

### 4.3. RNA-Seq Transcript Analysis

Total RNA samples were prepared by the LiCl method [17] from three-week old seedlings (in the 35S:A9 experiment), or from mature seeds imbibed for 24 h under dark (in the DS10:A9 and DS10:A4a experiments). For sequencing, which was performed twice for each RNA sample, we used in each case equimolecular mixtures of four, replicate RNA samples were prepared from corresponding pairs of transgenic (T: 35S:A9, DS10:A9 or DS10:A4a), and sibling non-transgenic (NT) lines. Library preparation, sequencing on an Illumina HiSeq 4000 sequencer using 100-bp-paired-end reads protocol, and downstream analyses were performed by BGI Co., Ltd. (Shenzhen, China). The Agilent 2100 Bioanalyzer (Agilent Technologies, Santa Clara, USA) and ABI StepOnePlus Real-Time PCR were used in the quantification and in qualification of the sample libraries. Sequenced reads were trimmed to remove adaptor sequences and masked to remove low-complexity or low-quality sequences. After filtering, the sequenced “clean reads” for each sample (at least 4.44 Gb bases) were mapped to a *Nicotiana tabacum* reference genome [34] using HISAT (v0.1.6-beta). Mapping ratios were around 90% in all cases. CuffCompare (v2.2.1) was used to compare reconstructed transcripts to reference annotation. After reconstructing transcripts, they were merged with the reference *Nicotiana tabacum* transcriptome [35] to get a complete reference to be used in gene expression analyses. Clean reads were then mapped to this reference using Bowtie2 (v2.2.5). Mapping ratios were larger than 70% in all cases. Gene expression levels for each sample were calculated with RSEM (v1.2.12) with default parameters. Using PoissonDis (Poisson Distribution) as described before [36], the expression results were analyzed to detect Differentially Expressed Genes (DEG) between the corresponding pairs of NT and T samples. We considered genes with a Fold Change (Log2) > 0.6 as DEG. For the additional comparisons between the DEG obtained from the different experiments, Venn diagrams were generated using Venny.

### 4.4. Protein Accumulation Gel Blot Analyses

Protein electrophoresis and western blot assays were performed as described previously [4,17]. Total protein was extracted in 2 × Laemmli buffer, the samples were quantified by Bradford (Biorad), and 30 µg of total protein was loaded per line in a SDS-PAGE gel at 8% (*w/v*) polyacrylamide. For western detection, the samples were transferred to Hybond-P membrane (Amersham Biosciences, Little Chalfont, UK) after electrophoresis. The membrane was incubated sequentially with primary antibodies anti-CRY1 (19) and anti-Actin (ab197345) from Abcam (Cambrigde, UK) at 1/5000 dilution, and the secondary antibody Goat anti-rabbit IgG HRP conjugated (AS09602) from Agrisera AB (Vännäs, Sweden) was used at a dilution of 1/25,000. The immunoreaction was detected using the Agrisera ECL SuperBright system (AS16 ECL-S-100).

### 4.5. Seed Deterioration

For fast deterioration assays (Basal Thermotolerance Assays, BTA), dry seeds were imbibed in water under controlled conditions and then subjected to a short incubation at 50 °C [4,7]. For that purpose, 30 mg of dry seeds (approx. 400–500 seeds) were rehydrated by sequential incubation in 1 mL of the sterilizing solution (1% (*v/v*) sodium hypochlorite) for 20 min and then washed several times with sterile water, followed by incubation in 1 mL of sterile water at 25 °C for 40 min. The rehydrated seeds were then incubated for 3 h at 50 °C in a water bath in direct contact with 1 mL of sterile water. At this step, the seeds reached approximately 40% moisture content. Germination was then scored by placing individual seeds at defined points in grids drawn of Petri dishes with 1 × Murashige and Skoog basal medium containing 3% (*w/v*) sucrose and 0.8% (*w/v*) phytoagar. The dishes were incubated at 25 °C (light, 16 h) and 20 °C (dark, 8 h) for 15 days.

### 4.6. Light Treatments and Quantification of Hypocotyl Growth Reduction and Seedling Greening

Light treatments for 6 h or 16 h (as indicated in the figures) were performed at 100 μmol·m^−2^·s^−1^ fluence intensity for white light, or at 20 μmol m^−2^·s^−1^ for blue light (440 nm). These treatments were performed at 23 °C in a FITOCLIMA (model 1200 BIO) LED cabinet (Aralab, Spain). Hypocotyl length measurements were performed on scanned images using the Image J software, essentially as described [17]. Seedling greening assays and the extraction and spectroscopic quantification of chlorophyllide, total chlorophyll, and carotenoids were also as previously described [17]. Because blue-light treatments prevented seed germination in our experimental conditions, germination was allowed to proceed under darkness for 3 days before the blue-light treatment. Controls were kept under darkness for 10 days.

### 4.7. Statistics

Differences between the transgenic and control groups of sibling material (seeds or seedlings) were tested using analysis of variance (ANOVA). For comparisons of germination after BTA (involving a temporal response), we used repeated measures ANOVA. Procedures for the statistical treatment have described in depth previously [4,5].

## Supplementary Materials

The following are available online at https://www.mdpi.com/2223-7747/9/3/335/s1. Table S1. The 4755 genes consistently upregulated by A9 in the RNA-seq experiments (see Figure 3A). Table S2. The A9up gene examples mentioned in the text. Table S3. The 13618 genes upregulated in the DS10:A4a RNA-seq experiment (see Figure 3B). Table S4. The subset of 1320 A9up genes co-activated by A9 and A4a (see Figure 3B). Table S5. The 16011 genes downregulated in the DS10:A4a RNA-seq experiment (see Figure 3C). Table S6. The subset of 1298 A9up genes downregulated in the DS10:A4a RNA-seq experiment (see Figure 3C). Table S7. Statistical comparisons mentioned in the text or in the Figures. The RNA-seq datasets supporting the conclusions of this article are available in the GEO repository under accession number GSE140837.

## References

[1] Scharf K.D., Berberich T., Ebersberger I., Nover L. (2012). The plant heat stress transcription factor (Hsf) family: Structure, function and evolution. Biochim. Biophys. Acta.

[2] Guo M., Liu J.H., Ma X., Luo D.X., Gong Z.H., Lu M.H. (2016). The Plant Heat Stress Transcription Factors (HSFs): Structure, Regulation, and Function in Response to Abiotic Stresses. Front. Plant Sci..

[3] Almoguera C., Rojas A., Diaz-Martin J., Prieto-Dapena P., Carranco R., Jordano J. (2002). A seed-specific heat-shock transcription factor involved in developmental regulation during embryogenesis in sunflower. J. Biol. Chem..

[4] Prieto-Dapena P., Castaño R., Almoguera C., Jordano J. (2006). Improved resistance to controlled deterioration in transgenic seeds. Plant Physiol..

[5] Prieto-Dapena P., Castaño R., Almoguera C., Jordano J. (2008). The ectopic overexpression of a seed-specific transcription factor, HaHSFA9, confers tolerance to severe dehydration in vegetative organs. Plant J..

[6] Almoguera C., Prieto-Dapena P., Personat J.M., Tejedor-Cano J., Lindahl M., Diaz-Espejo A., Jordano J. (2012). Protection of the Photosynthetic Apparatus from Extreme Dehydration and Oxidative Stress in Seedlings of Transgenic Tobacco. PLoS ONE.

[7] Tejedor-Cano J., Prieto-Dapena P., Almoguera C., Carranco R., Hiratsu K., Ohme-Takagi M., Jordano J. (2010). Loss of function of the HSFA9 seed longevity program. Plant Cell Environ..

[8] Tejedor-Cano J., Carranco R., Personat J.-M., Prieto-Dapena P., Almoguera C., Manuel Espinosa J., Jordano J. (2014). A Passive Repression Mechanism that Hinders Synergic Transcriptional Activation by Heat Shock Factors Involved in Sunflower Seed Longevity. Mol. Plant.

[9] Carranco R., Espinosa J.M., Prieto-Dapena P., Almoguera C., Jordano J. (2010). Repression by an auxin/indole acetic acid protein connects auxin signaling with heat shock factor-mediated seed longevity. Proc. Natl. Acad. Sci. USA.

[10] Personat J.M., Tejedor-Cano J., Prieto-Dapena P., Almoguera C., Jordano J. (2014). Co-overexpression of two Heat Shock Factors results in enhanced seed longevity and in synergistic effects on seedling tolerance to severe dehydration and oxidative stress. BMC Plant Biol..

[11] Pogson B.J., Ganguly D., Albrecht-Borth V. (2015). Insights into chloroplast biogenesis and development. Biochimica et Biophysica Acta.

[12] Franklin K.A., Quail P.H. (2010). Phytochrome functions in Arabidopsis development. J. Exp. Bot.

[13] Chen M., Chory J. (2011). Phytochrome signaling mechanisms and the control of plant development. Trends Cell Biol..

[14] Wang H., Wang H. (2015). Phytochrome signaling: Time to tighten up the loose ends. Mol. Plant.

[15] Chaves I., Pokorny R., Byrdin M., Hoang N., Ritz T., Brettel K., Essen L.O., Van Der Horst G.T.J., Batschauer A., Ahmad M. (2011). The cryptochromes: Blue light photoreceptors in plants and animals. Annu. Rev. Plant Biol..

[16] Christie J.M. (2007). Phototropin blue-light receptors. Annu. Rev. Plant Biol..

[17] Prieto-Dapena P., Almoguera C., Personat J.M., Merchan F., Jordano J. (2017). Seed-specific transcription factor HSFA9 links late embryogenesis and early photomorphogenesis. J. Exp. Bot.

[18] Rojas A., Almoguera C., Carranco R., Scharf K.D., Jordano J. (2002). Selective Activation of the Developmentally Regulated *Hahsp17.6G1* Promoter by Heat Stress Transcription Factors. Plant Physiol..

[19] Lin C., Ahmad M., Gordon D., Cashmore A.R. (1995). Expression of an Arabidopsis cryptochrome gene in transgenic tobacco results in hypersensitivity to blue, UV-A, and green light. Proc. Natl. Acad. Sci. USA.

[20] Poppe C., Sweere U., Drumm-Herrel H., Schäfer E. (1998). The blue light receptor cryptochrome 1 can act independently of phytochrome A and B in Arabidopsis thaliana. Plant J..

[21] Kotak S., Port M., Ganguli A., Bicker F., Von Koskull-Doring P. (2004). Characterization of C-terminal domains of Arabidopsis heat stress transcription factors (Hsfs) and identification of a new signature combination of plant class A Hsfs with AHA and NES motifs essential for activator function and intracellular localization. Plant J..

[22] Hiratsu K., Matsui K., Koyama T., Ohme-Takagi M. (2003). Dominant repression of target genes by chimeric repressors that include the EAR motif, a repression domain, in Arabidopsis. Plant J..

[23] Rajjou L., Lovigny Y., Groot S.P., Belghazi M., Job C., Job D. (2008). Proteome-wide characterization of seed aging in Arabidopsis: A comparison between artificial and natural aging protocols. Plant Physiol..

[24] Sano N., Rajjou L., North H.M., Debeaujon I., Marion-Poll A., Seo M. (2016). Staying Alive: Molecular Aspects of Seed Longevity. Plant Cell Physiol..

[25] Leprince O., Buitink J. (2010). Desiccation tolerance: From genomics to the field. Plant Sci..

[26] Finch-Savage W.E., Bassel G.W. (2016). Seed vigour and crop establishment: Extending performance beyond adaptation. J. Exp. Bot.

[27] Friedberg J.N., Bowley S.R., McKersie B.D., Gurley W.B., Czarnecka-Verner E. (2006). Isolation and characterization of class A4 heat shock transcription factor from alfalfa. Plant Sci..

[28] Baniwal S.K., Chan K.Y., Scharf K.D., Nover L. (2007). Role of heat stress transcription factor HsfA5 as specific repressor of HsfA4. J. Biol. Chem..

[29] Shim D., Hwang J.U., Lee J., Lee S., Choi Y., An G., Martinoia E., Lee Y. (2009). Orthologs of the Class A4 Heat Shock Transcription Factor HsfA4a Confer Cadmium Tolerance in Wheat and Rice. Plant Cell.

[30] Lang S., Liu X., Xue H., Li X., Wang X. (2017). Functional characterization of BnHSFA4a as a heat shock transcription factor in controlling the re-establishment of desiccation tolerance in seeds. J. Exp. Bot..

[31] Sanchez-Lamas M., Lorenzo C.D., Cerdan P.D. (2016). Bottom-up Assembly of the Phytochrome Network. PLoS Genet.

[32] Hloušková P., Bergougnoux V. (2016). A subtracted cDNA library identifies genes up-regulated during PHOT1-mediated early step of de-etiolation in tomato (*Solanum lycopersicum* L.). BMC Genom..

[33] Bourbousse C., Mestiri I., Zabulon G., Bourge M., Formiggini F., Koini M.A., Brown S.C., Fransz P., Bowler C., Barneche F. (2015). Light signaling controls nuclear architecture reorganization during seedling establishment. Proc. Natl. Acad. Sci. USA.

[34] *Nicotiana tabacum* Reference Genome. ftp://ftp.solgenomics.net/genomes/Nicotiana_tabacum/sierro_et_al_2014/assembly/Ntab-BX_AWOK-SS.fa.gz.

[35] *Nicotiana tabacum* Transcriptome. ftp://ftp.solgenomics.net/tobacco_genome/sierro_et_al_2014/annotation/Ntab-BX_AWOK-SS_Basma.mrna.annot.fna.

[36] Audic S., Claverie J.M. (1997). The significance of digital gene expression profiles. Genome Res..

