# Supplementary material for: Heat Stress Factors Expressed during Seed Maturation Differentially Regulate Seed Longevity and Seedling Greening
Source: Plants (Basel). 2020 Mar 6;9(3):335. doi: 10.3390/plants9030335 (PMC7154816; doi:10.3390/plants9030335)
Supplement: Supplementary file 1 [file plants-09-00335-s001.zip › Table S7.docx]

**Table S7**

| **Comparison** | **Statistics** |
| --- | --- |
| PHYA + A9 to PHYA + A9 + A4 (Fig. 1) | *F*= 32.004, *P*< 0.0001 |
| Chl, A9 to A9/A4a, 16h under white light (Fig. 2) | *F*= 5.43, *P*= 0.033 |
| Car, A9 to A9/A4a, 16h under white light (Fig. 2) | *F*= 9.48, *P*= 0.0065 |
| Chlide, A9 to A9/A4a, 16h under white light (Fig. 2) | *F*= 4.57, *P*= 0.048 |
| Hypocotyl length (mm), Bc, NT to 35S:A9 (Fig. 5) | *F*= 1097.6, *P*< 0.0001 |
| Hypocotyl length (mm), Dark, NT to 35S:A9 (Fig. 5) | *F*= 1399, *P*< 0.0001 |
| Hypocotyl length (%), Bc, NT to 35S:A9 (Fig. 5) | *F*= 213.7, *P*< 0.0001 |
| Germination, NT to A9M1 (Fig. 6) | *F*= 9.04, *P*= 0.011 |
| Chl, % NT, A9M1 to A9M3, 6h under white light (Fig. 7) | *F*= 0.283, *P*= 0.598 |
| Chl, % NT, A9M1 to A9M3, 16h under white light (Fig. 7) | *F*= 0.079, *P*= 0.78 |
| Car, % NT, A9M1 to A9M3, 6h under white light (Fig. 7) | *F*= 0.103, *P*= 0.751 |
| Car, % NT, A9M1 to A9M3, 6h under white light (Fig. 7) | *F*= 0.016, *P*= 0.899 |
